# Supplementary material for: Enhancing prosthetic vision by upgrade of a subretinal photovoltaic implant in situ
Source: Nat Commun. 2025 Mar 22;16:2820. doi: 10.1038/s41467-025-58084-y (PMC11928519; doi:10.1038/s41467-025-58084-y)

Supplementary figures for:

Enhancing Prosthetic Vision by Upgrade of a Subretinal Photovoltaic Implant in situ

Mohajeet B. Bhuckory[1,2,@], Nicharee Monkongpitukkul[2,3] *, Andrew Shin[4] *, Anna Kochnev Goldstein[5], Nathan Jensen[5], Sarthak V Shah[2], Davis Pham-Howard[1,2], Emma Butt[6], Roopa Dalal[2], Ludwig Galambos[1], Keith Mathieson[6], Theodore Kamins[5], and Daniel Palanker[1,2]

[1] Hansen Experimental Physics Laboratory, Stanford University, Stanford, CA 94303, USA

[2] Department of Ophthalmology, Stanford University, Stanford, CA, USA

[3] Department of Ophthalmology, Faculty of Medicine, Prince of Songkla University, Thailand

[4] Department of Material Science, Stanford University, Stanford, CA, USA

[5] Department of Electrical Engineering, Stanford University, Stanford, CA, USA

[6] Department of Physics, University of Strathclyde, Glasgow, Scotland, UK

*Contributed equally

[@] Corresponding author – Mohajeet B Bhuckory; 300 Pasteur drive, Dept of Ophthalmology, Stanford university, CA 94303, USA; bhuckory@stanford.edu

Supplementary Fig. 1. Microglial cells (green) labelled with IBA1 show a similar distribution of the immune cells through the retina when the control (A; n=3) is compared to the extracted implant retina (B-D; n=3 per group). Scale bar is 80 µm. Calbindin (magenta; asterisk) labelled horizontal cells are sparsely present in RCS control (E; n=4) and explanted implant retina (F-H; n=4 per group), with a few disorderly dendrites visible. Scale bar is 100 µm. (I) GFAP quantification of the IPL to subretinal space, excluding the endfeet of Müller cells (one-way ANOVA; Turkey's post hoc; p=0.05). (J) IBA1 quantification of the INL to the subretinal space to assess the accumulation of microglial cells after extraction surgery (one-way ANOVA; Turkey's post hoc; p=0.08). N numbers represent individual experiments with biological replicates, all yielding similar results. Data are presented as mean values and error bars +/- SEM INL- inner nuclear layer; GCL – ganglion cell layer; RPE – retinal pigment epithelium.

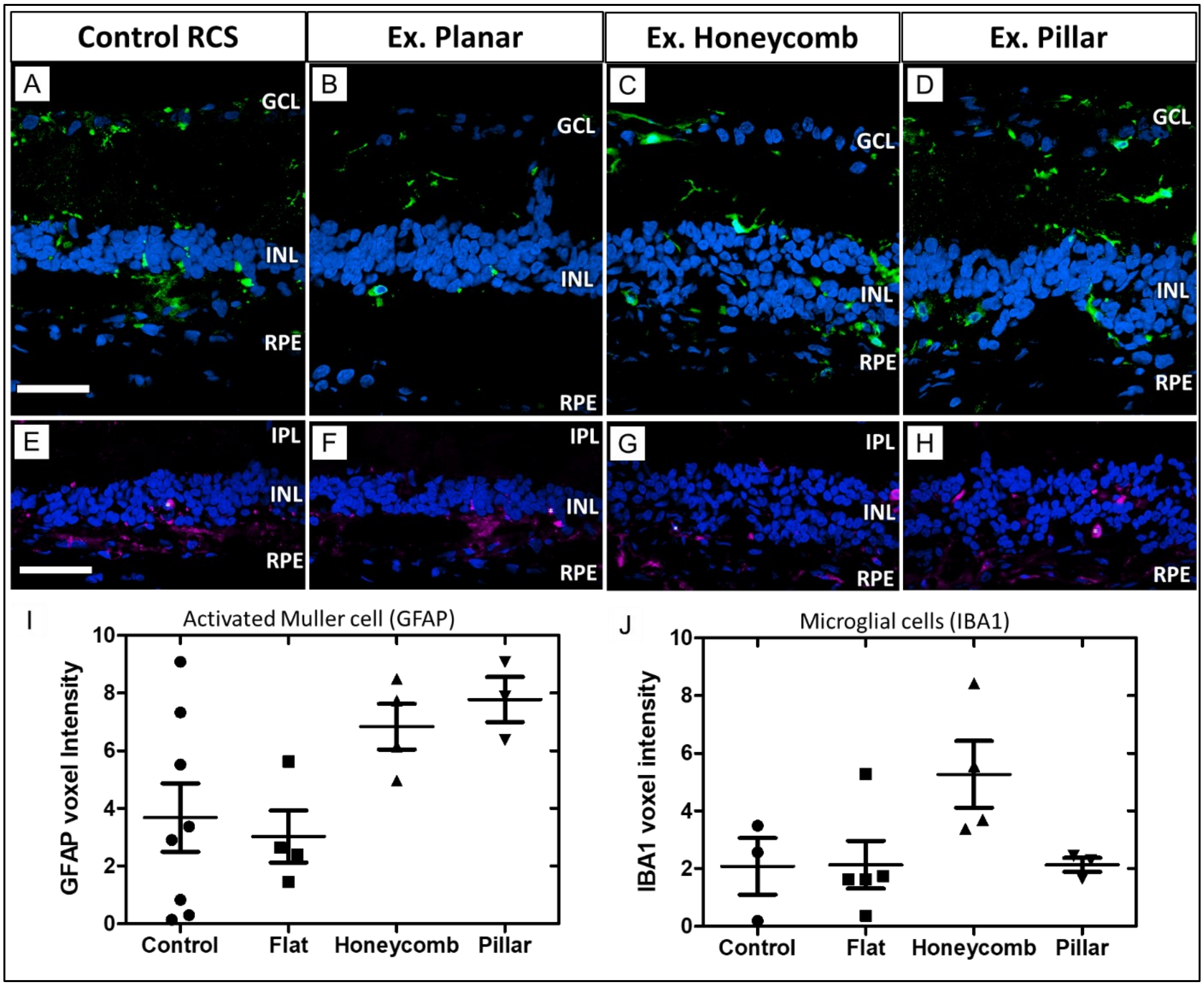

Supplementary Figure. 2. Toluidine blue stained histological section 6 weeks post-explantation (n=3). A thick acellular layer (yellow arrow) develops in the subretinal space (red dotted line marks the RPE/choroid boundary). The subretinal fibrosis is localized to the area where the implant was (yellow dotted lines mark the location of primary implant). Scale bar 150 μm.

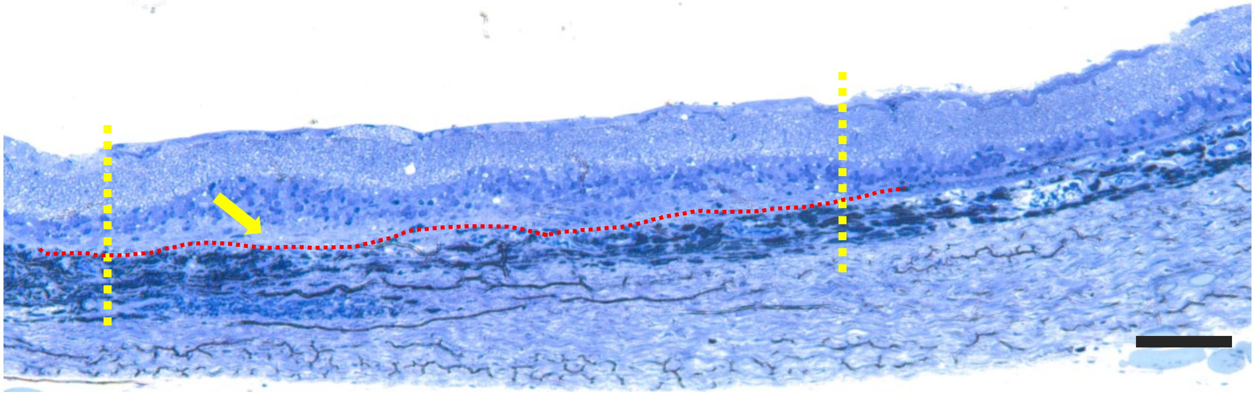

Supplementary Figure. 3. OCT image of an RCS control retina before surgery (n= 12). The lens occupies the majority of the anterior chamber, leaving approximately 1 mm of space for retinal detachment, tool insertion and subretinal manipulations.

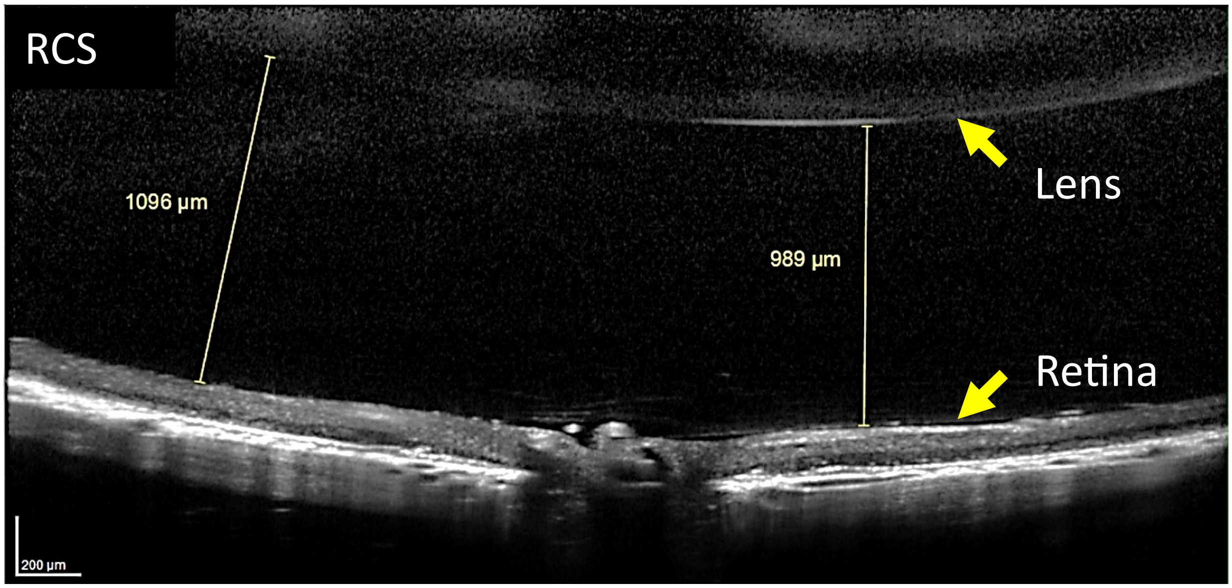

Supplement: Supplementary file 1 — Supplementary Information [file 41467_2025_58084_MOESM1_ESM.pdf]
