# Supplementary material for: Enhancing prosthetic vision by upgrade of a subretinal photovoltaic implant in situ
Source: Nat Commun. 2025 Mar 22;16:2820. doi: 10.1038/s41467-025-58084-y (PMC11928519; doi:10.1038/s41467-025-58084-y)
Supplement: Supplementary file 2 — Description of Additional Supplementary Information [file 41467_2025_58084_MOESM2_ESM.docx]

**Description of Additional Supplementary Files**

File Name: Supplementary Video 1

Description: Implant removal and upgrade. The key surgical steps of the removal of a PRIMA 100 µm implant and replacement with a monopolar (MP) 22 µm implant, including: retinal detachment from the PRIMA implant by BSS injection, grabbing the implant and dragging it in the subretinal space, extraction of the PRIMA implant through the 1.5 mm incision in the sclera, coating the MP device with viscoelastic gel, inserting the implant through the same incision, and placing the new device in the same location as the PRIMA.
