# Supplementary material for: Enhancing prosthetic vision by upgrade of a subretinal photovoltaic implant in situ
Source: Nat Commun. 2025 Mar 22;16:2820. doi: 10.1038/s41467-025-58084-y (PMC11928519; doi:10.1038/s41467-025-58084-y)
Supplement: Supplementary file 4 — Reporting Summary [file 41467_2025_58084_MOESM4_ESM.pdf]

nature portfolio

Corresponding author(s): Mohajeet B bhuckory

Last updated by author(s): 1/24/2025

Reporting Summary

Nature Portfolio wishes to improve the reproducibility of the work that we publish. This form provides structure for consistency and transparency in reporting. For further information on Nature Portfolio policies, see our Editorial Policies and the Editorial Policy Checklist.

Statistics

For all statistical analyses, confirm that the following items are present in the figure legend, table legend, main text, or Methods section.

n/a	Confirmed	
☐	☒	The exact sample size (*n*) for each experimental group/condition, given as a discrete number and unit of measurement
☐	☒	A statement on whether measurements were taken from distinct samples or whether the same sample was measured repeatedly
☐	☒	The statistical test(s) used AND whether they are one- or two-sided<br>*Only common tests should be described solely by name; describe more complex techniques in the Methods section.*
☐	☒	A description of all covariates tested
☐	☒	A description of any assumptions or corrections, such as tests of normality and adjustment for multiple comparisons
☐	☒	A full description of the statistical parameters including central tendency (e.g. means) or other basic estimates (e.g. regression coefficient) AND variation (e.g. standard deviation) or associated estimates of uncertainty (e.g. confidence intervals)
☐	☒	For null hypothesis testing, the test statistic (e.g. *F*, *t*, *r*) with confidence intervals, effect sizes, degrees of freedom and *P* value noted<br>*Give P values as exact values whenever suitable.*
☒	☐	For Bayesian analysis, information on the choice of priors and Markov chain Monte Carlo settings
☒	☐	For hierarchical and complex designs, identification of the appropriate level for tests and full reporting of outcomes
☒	☐	Estimates of effect sizes (e.g. Cohen's *d*, Pearson's *r*), indicating how they were calculated

Our web collection on statistics for biologists contains articles on many of the points above.

Software and code

Policy information about availability of computer code

Data collection	Espion E3 system (Diagnosys LLC), HEYEX v.1.12.40.
Data analysis	MATLAB (vR2017a and vR2020a). GraphPad Prism 10 , R version 4.x., Python 3.8.8, HEYEX v.1.12.40.<br>Codes for VEP data processing are available from Zenodo database at https://doi.org/10.5281/zenodo.14728729 and for implant reconstitution after confocal imaging at https://doi.org/10.5281/zenodo.14728769

For manuscripts utilizing custom algorithms or software that are central to the research but not yet described in published literature, software must be made available to editors and reviewers. We strongly encourage code deposition in a community repository (e.g. GitHub). See the Nature Portfolio guidelines for submitting code & software for further information.

Data

Policy information about availability of data

All manuscripts must include a data availability statement. This statement should provide the following information, where applicable:

- Accession codes, unique identifiers, or web links for publicly available datasets
- A description of any restrictions on data availability
- For clinical datasets or third party data, please ensure that the statement adheres to our policy

The data that support the findings of this study are freely available on the Zenodo database under doi https://doi.org/10.5281/zenodo.14728792. All other relevant data supporting the key findings of this study are available within the article and its Supplementary Information files.

Additionally, the source data file has been uploaded.

Research involving human participants, their data, or biological material

Policy information about studies with human participants or human data. See also policy information about sex, gender (identity/presentation), and sexual orientation and race, ethnicity and racism.

Reporting on sex and gender	Equal number of male and female rats were used in all studies.
Reporting on race, ethnicity, or other socially relevant groupings	N/A
Population characteristics	N/A
Recruitment	N/A
Ethics oversight	All experimental protocols received approval from the Administrative Panel on Laboratory Animal Care (APLAC) at Stanford and were executed following institutional guidelines. The procedures adhered to the Statement for the Use of Animals in Ophthalmic and Vision Research, as outlined by the Association for Research in Vision and Ophthalmology (ARVO).

Note that full information on the approval of the study protocol must also be provided in the manuscript.

Field-specific reporting

Please select the one below that is the best fit for your research. If you are not sure, read the appropriate sections before making your selection.

[x] Life sciences [ ] Behavioural & social sciences [ ] Ecological, evolutionary & environmental sciences

For a reference copy of the document with all sections, see nature.com/documents/nr-reporting-summary-flat.pdf

Life sciences study design

All studies must disclose on these points even when the disclosure is negative.

Sample size	For anatomical studies, a total of N = 38 animals were implanted subretinally with different types of arrays (15 planar, 17 honeycomb and 6 pillar implants), and the devices were explanted 6 weeks later. Extraction studies, OCT and immunohistochemistry had 4 animals per group. Reimplantation studies had 8 controls, 6 primary implant and 5 reimplanted implants were implanted with a PRIMA 100 µm chip for 6 weeks and the array was replaced with a 22 µm planar implant and monitored for up to 6 months. VEP studies had 4 animals per group. Experiments maintained equal numbers of male and female rats.
Data exclusions	No data were excluded from analysis
Replication	We have measured each independent sample multiple times (with specific n numbers provided as biological replicates) to ensure the reproducibility of stated findings.
Randomization	The animals participating this study were randomly selected from our colony. The choice of implanted device was also randomized.
Blinding	Blinding was not relevant nor possible in this study. Researchers have to visualize the implanted devices during surgery and measurements, and thus know the groups. However, human biases were avoided through blinding between image acquisition and processing.

Reporting for specific materials, systems and methods

We require information from authors about some types of materials, experimental systems and methods used in many studies. Here, indicate whether each material, system or method listed is relevant to your study. If you are not sure if a list item applies to your research, read the appropriate section before selecting a response.

Materials & experimental systems

n/a	Involved in the study	
[ ]	[x]	Antibodies
[x]	[ ]	Eukaryotic cell lines
[x]	[ ]	Palaeontology and archaeology
[ ]	[x]	Animals and other organisms
[x]	[ ]	Clinical data
[x]	[ ]	Dual use research of concern
[x]	[ ]	Plants

Methods

n/a	Involved in the study	
[x]	[ ]	ChIP-seq
[x]	[ ]	Flow cytometry
[x]	[ ]	MRI-based neuroimaging

Antibodies

Antibodies used	1) 1:500 of goat raised glial fibrillary acidic protein primary antibody (GFAP; SC-6170; Santa Cruz Biotechnologies, Santa Cruz, CA), 2) 1:100 Mouse anti-glutamine synthetase (GS; NBP2-43646; Novus, CA), 3), 1:400 rabbit raised IBA1 antibody (WAKO, Japan), 1:100 mouse anti calbindin antibody (Swant; CB300; CA). and 1) 1:400 donkey raised anti goat, Alexa Fluor (AF) 594 conjugated secondary antibody (A-11058; Thermo Fisher Scientific, Rockford, IL), 2) 1:500 donkey anti-rabbit AF488 (A-21206; Thermo Fisher Scientific, Rockford, IL), 3) donkey anti mouse CY3 conjugated secondary antibody (715-165-150; Jackson labs, USA)
Validation	The antibodies used marked visually distinguishable whole cell structures that validated the specificity of the antibodies. The manufacturers provided fact sheets about the antibodies and we have previously published results with the same antibodies: https://doi.org/10.1073/pnas.2307380120

Animals and other research organisms

Policy information about studies involving animals; ARRIVE guidelines recommended for reporting animal research, and Sex and Gender in Research

Laboratory animals	Rats: Royal College of Surgeons (RCS-p+/LavRrrc; RRID:RRRC_00315, breeders purchased from RRRC, Missouri University; colony maintained at the Stanford Animal Facility) rats with a genetic mutation in MERTK gene; male and female, 6 to 9 months old
Wild animals	N/A
Reporting on sex	Findings are not dependent upon animal sex.
Field-collected samples	N/A
Ethics oversight	Stanford Administrative Panel on Laboratory Animal Care (APLAC)

Note that full information on the approval of the study protocol must also be provided in the manuscript.

Plants

Seed stocks	N/A
Novel plant genotypes	N/A
Authentication	N/A
